# Effect of Wind Farm Noise on Local Residents’ Decision to Adopt Mitigation Measures

**DOI:** 10.3390/ijerph14070753

**Published:** 2017-07-11

**Authors:** Anabela Botelho, Pedro Arezes, Carlos Bernardo, Hernâni Dias, Lígia M. Costa Pinto

**Affiliations:** 1 Department of Economics, Management, Industrial Engineering, and Tourism, University of Aveiro, and GOVCOPP, 3810-193 Aveiro, Portugal; anabela.botelho@ua.pt; 2 ALGORITMI Research Center, University of Minho, 4800-058 Guimarães, Portugal; 3 Institute of Polymers and Composites/I3N, University of Minho, 4800-058 Guimarães, Portugal; cbernardo@dep.uminho.pt; 4 PIEP-Innovation in Polymer Engineering, 4800-058 Guimarães, Portugal; hernadias@gmail.com; 5 NIMA-Department of Economics, University of Minho, 4710-057 Braga, Portugal; pintol@eeg.uminho.pt

**Keywords:** wind farms, noise annoyance, mitigation measures, sound pressure levels, environmental impacts

## Abstract

Wind turbines’ noise is frequently pointed out as the reason for local communities’ objection to the installation of wind farms. The literature suggests that local residents feel annoyed by such noise and that, in many instances, this is significant enough to make them adopt noise-abatement interventions on their homes. Aiming at characterizing the relationship between wind turbine noise, annoyance, and mitigating actions, we propose a novel conceptual framework. The proposed framework posits that actual sound pressure levels of wind turbines determine individual homes’ noise-abatement decisions; in addition, the framework analyzes the role that self-reported annoyance, and perception of noise levels, plays on the relationship between actual noise pressure levels and those decisions. The application of this framework to a particular case study shows that noise perception and annoyance constitutes a link between the two. Importantly, however, noise also directly affects people’s decision to adopt mitigating measures, independently of the reported annoyance.

## 1. Introduction

Wind has been used as a power source almost since the rise of human civilization. Later, in the most advanced economies, its intermittent and (then) unpredictable nature, made it uncompetitive with other power sources, namely those based on steam. Recently, due to technological developments, its use, especially for electricity generation, has been increasing worldwide [1]. Nowadays, wind power is considered to be an environmentally-friendly energy source, technologically mature and economically competitive.

However, the installation of wind turbines (WTs) has consequences in the use of land and may have other negative side effects. Amongst these, the effect of noise on human health and annoyance is of paramount importance. Although the measurement of annoyance is highly subjective, the concept can be understood as a cause of irritation or vexation; a nuisance. A good definition is provided by Guski et al. [2], namely “a multifaceted concept, covering mainly immediate behavioral noise effects aspects, like disturbance and interfering with intended activities”. In fact, WT noise can be easily perceived (and be an annoyance) even for low sound pressure levels, making it generally incongruous with background noise [3]. As a consequence, it may be a problem for local communities, and one of the reasons they often object to the installation of new wind farms in their vicinity. Indeed, after lack of knowledge on environmental issues related to renewable energies, this is the second major cause of opposition to the implementation of such projects [4]. For example, evidence rendered in a public hearing in New Zealand revealed that most people living close to WT are able to perceive the noise generated by them, although, as expected, the effect decreased with distance [5]. Another disclosure of this hearing was that people were more annoyed with turbine noise during the night period, although its measured characteristics were quite similar to those determined during the day. As a consequence, some respondents decided to sleep with earplugs or even to improve their homes’ sound insulation. These findings are consistent with other studies reporting that people living near wind farms complain of a variety of negative physiological and psychological symptoms [6]. In certain cases, these symptoms are sufficiently serious to force them to modify their homes (e.g., implementation of sound insulation measures) to reduce the intrusive noise or even, in the extreme, to abandon their residences [7,8].

The literature on the effects of wind turbine noise on the health and annoyance of exposed people, which has grown significantly since 2000, has been the subject of various recent reviews. The first one, by Jeffery et al. [9], concluded that there is sufficient evidence to believe that people exposed to audible WT noise experience negative effects in their physical, mental and social well-being and that those symptoms can result from the ensuing annoyance. Schmidt and Klokker [10] also report that in the literature there is evidence of a dose-response relationship between wind turbine noise, sleep disturbance and possibly even psychological distress, linked to noise annoyance. A large study involving 1238 households in Southern Ontario and Prince Edward Island published in 2014 by the Government of Canada [11], also concluded that proximity to wind farms is associated with annoyance and, less consistently, with sleep disturbance and poorer quality of life. Onakpoya et al. [12] reviewed published data on the association between exposure to wind turbine noise, sleep disturbance and quality of life. Globally, they concluded that there is some evidence for a causal relationship between increased odds of annoyance and sleep problems, although individual attitudes could influence the type of response. On the other hand, an extensive and recently published study commissioned by the Australian government on the impact of wind farms on human health concluded that there is no reliable or consistent evidence that proximity to wind farms, or wind farm noise directly causes health effects [13]. That conclusion is partially contradicted by a study in Canada [14] that found that there is sufficient evidence to establish a direct causal relationship between exposure to wind turbine noise and annoyance but not with sleep disturbance. However, an indirect (via annoyance) causal relationship might exist with the latter. A recent study by Jalali et al. [15], using a prospective cohort design, presented noise and sleep measurements done before and after installation of WT. It showed that participants reported poorer sleep quality if they had a previous negative attitude to WT, if they were concerned with property devaluation, or if they could see turbines from their properties. Thus, although the controversy about the effects of WT noise on human health is far from over, the current overall literature seems to indicate that it can affect, to a greater or lesser extent, the wellness of the people exposed. The literature also seems to suggest that annoyance plays a mediator role in that relationship.

Furthermore, in addition to probable adverse health effects and reduced quality of life, exposed individuals face the financial burden of having to take measures to solve, or at least mitigate, the effects of the unwanted sound caused by WT. While such financial burden may constitute an important component of the external economic costs borne by those living close to wind farms, no study to date has empirically evaluated the actual effect of measured WT noise on individuals’ necessity to modify their homes. Thus, in the present study, we address this issue by proposing a conceptual framework relating those sound levels with individual homes’ noise-abatement interventions, and assessing the role that self-reported annoyance (a proxy for noise perception), both at indoors and outdoors settings, plays in that relationship. This is done by applying structural modeling and estimation techniques that are appropriate to empirically test the proposed framework given the type of data collected. The described analysis was restricted to noise exposed populations and did not include any comparison with other populations not exposed to WT noise. In fact, it was assumed that in rural settings such as the one under study, far from alternative strong noise sources, there is no clear need to adopt any noise-abatement interventions. Conversely, the study considers subjects that decided to take noise abatement measures, and those that did not, the latter providing a population control group.

The remainder of the paper is structured as follows: Section 2 presents the conceptual framework, outlines the procedures used in data collection, and provides details about the econometric specification and estimation strategy. Section 3 reports and discusses the empirical results, and Section 4 concludes.

## 2. Materials and Methods

### 2.1. Conceptual Framework

**Figure 1:**
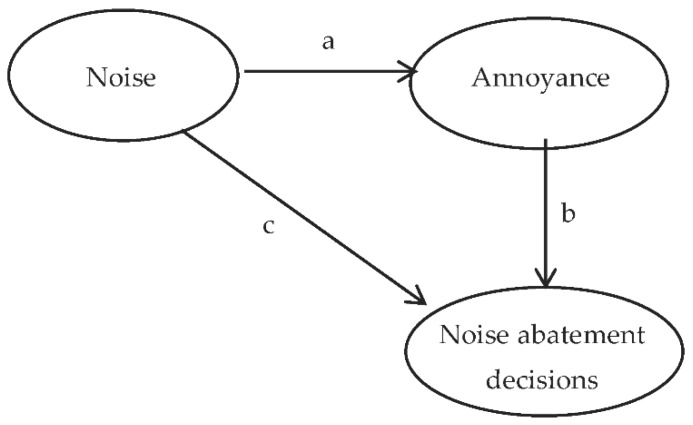
Exposure-response relationship for wind turbine noise.

Annoyance is the most commonly reported problem caused by wind turbine noise exposure. In severe forms it may affect individuals’ health and well-being thereby contributing to the burden of this environmental noise [16]. Thus, noise-induced annoyance has often been the primary response variable used in social surveys to evaluate the health effects of noise on people living close to WT, even if the relationship with noise characteristics are not yet well explained or understood [17]. While the association between wind turbine noise and annoyance is generally accepted, controversy remains about the importance of this association. If acoustic factors such as sound pressure levels (SPL) only partly influence individuals’ annoyance response, their public health implications should be small. In addition, annoyance is a subjective psychological concept, and the fact that noise causes annoyance does not necessarily mean that it significantly impairs individuals’ health. Legally, there is case law evidence that environmental hazards need to attain a minimum level of disturbance (resulting in a sufficiently severe injury to individuals’ property, their private or family life) in order to justify authorities’ implementation of precautionary, protective, or compensatory measures [18]. Along with annoyance, revealed preference of individuals’ seeking health-related restoration or well-being by temporarily or permanently leaving their residences has also been identified as a criterion for diagnosis of “probable adverse health effects” in the vicinity of WT, the latter being singled out as a distinguishing factor between adverse health effects due to WT versus independent causes [19]. Thus, revealed preference information concerning individuals’ reactions to wind turbine noise allows a better assessment, not only of the causal relationship between noise and health/well-being outcomes, but also of the severity of the burden. Indeed, studies focusing on adverse event reports consider individuals’ willingness to spend resources on retrofitting their houses to reduce noise as an indication of both individuals’ conviction regarding the causal pathway between WT noise and disease and of the intensity of their noise-induced suffering [20]. Moreover, establishing a link between annoyance, an individual’s subjective feeling, and his actions to prevent it, may constitute a significant contribution to the acceptability of annoyance studies’ results. In line with these considerations, a conceptual framework relating noise exposure to the decision to take retrofitting measures is presented in Figure 1.

The conceptual framework presented in Figure 1 posits that the overall impact of wind turbine noise is quantified based on the relationship between noise dose and response, the latter measured by revealed preference information describing individuals’ willingness to spend resources on retrofitting their houses. Considering the diagram, there is also some direct effect between noise and individuals’ well-being in terms of annoyance. In turn, annoyance may lead to forced behavioral responses with the aim of decreasing the overall noise burden. Thus, the subjective appraisal of annoyance mediates the relationship between noise levels and the outcome, as represented by the indirect effect *b*. Whether noise annoyance, as subjectively evaluated, fully or only partly mediates the proposed exposure-response relationship depends on the significance of path *c*. If the entire noise effect goes through annoyance, then path *c* takes the value zero, and there is full mediation. If the noise level acts as an independent contributor to the revealed outcome variable, irrespective of annoyance, then the effect *c* will be nonzero, and there is partial mediation.

### 2.2. Data Collection

The present work was carried out in the Fafe Highlands (*Terras Altas de Fafe*) wind farm in the north of Portugal. This wind farm is located in the municipalities of Fafe and Celorico de Basto, in a mountain area, 851 m height on average. The farm is composed of 53 WT, corresponding to 106 MW of total installed capacity. The annual production is estimated at circa 210 GWh (for an equivalent of 2000 h of full load/year). The turbines are all dimensionally identical, with a 67 m height tower and a 87 m rotor diameter. A-weighted SPL measured near the facades of the houses located in the periphery of the farm were chosen as measuring standards. The results of these measurements were cross-checked with the answers to a questionnaire on noise perception collected from the residents of those houses.

#### 2.2.1. Data Collection Procedures

Considering the research objectives, the planned research was carried out according to the following protocol:

(1) Characterization of the location of wind farms relatively to nearby populations; this characterization was based on a specific typological classification, considering, amongst other factors, the visibility of the turbines;
(2) Direct measurement of the SPL in the selected areas, with a view to assess the acoustic impact of the wind farms. In the current study, noise levels were measured in different zones within the exposed villages, and the corresponding values were used as a proxy indicator (and not a precise noise dose or exposure level) of the noise exposure of the inhabitants of that same zone;
(3) Development of a questionnaire on noise perception based on previous ones developed by Pedersen and Waye [21] properly adapted to the Portuguese situation;
(4) Application of the questionnaire to the residents of chosen villages.

#### 2.2.2. Sound Measurements

**Figure 2:**
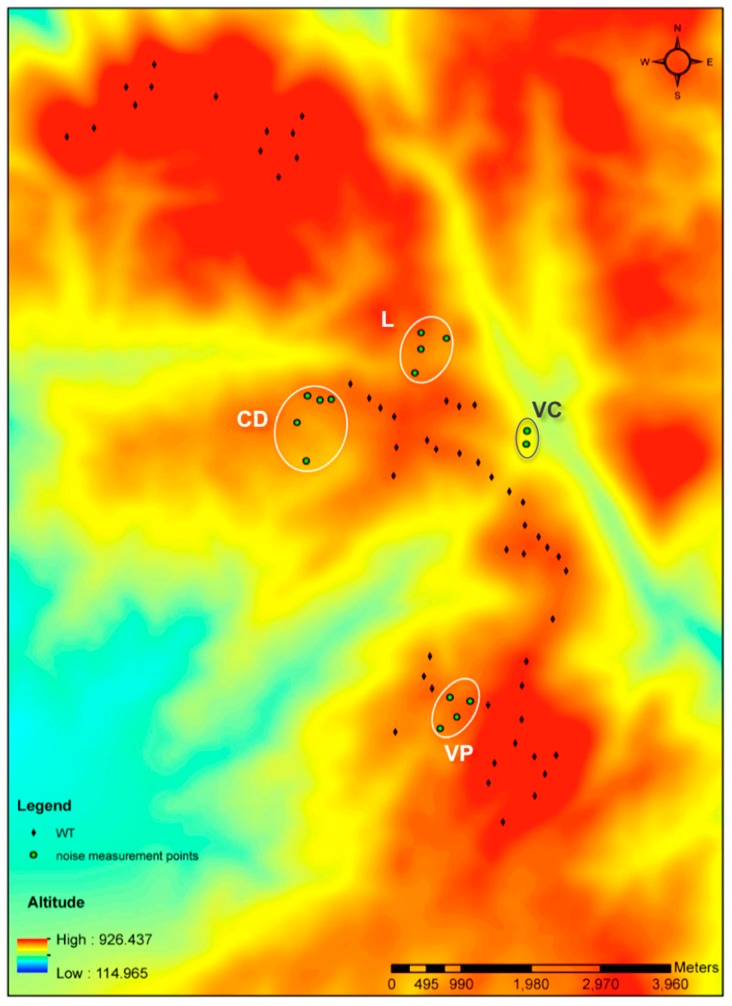
Map of the wind farm studied with the corresponding measurement locations (black dots: Wind Turbines; green circles: dwellings at the measurements’ locations, L: Lagoa, CD: Campo Dianteiro, VC: Várzea Cova, VP: Vila Pouca).

The sound measurements were carried out in the villages of Campo Dianteiro (CD), Lagoa (L), Várzea Cova (VC) and Vila Pouca (VP), all in the immediate vicinity of the wind farm (Figure 2).

Inspection of Figure 2 allows a rough estimate of the distance from the villages/dwellings to the WT, which ranges from 250 to 1000 m. In each village, two different areas were defined corresponding to a “high” and a “low” point (as most of the villages have a steep topography) and the measurements were grouped accordingly. The determinations were made using a sound level meter Bruel&Kjaer model 2260 type 1, equipped with a tripod. The equipment was positioned at a 1.2 m of height from the ground and no closer than 4.0 m from the facades, in order to avoid reflection effects. In each location, the ‘A’ frequency equivalent continuous sound pressure level (LAeq) was registered considering a 5 min measurement period, during which the background noise was monitored. This was necessary to avoid the inclusion of “external” irrelevant noise events not related to the WT. When one such event occurred, as for example, the sudden barking of a dog or the passing of a car, the measurement was stopped and the event eliminated. During the measurements, the wind speed was also assessed and registered and found to be consistently low, i.e., less than 2 m/s. It is also important to highlight that some sporadic impulsive characteristics were sometimes detected, mainly from the existent background noise, which seemed not to affect the WT registered overall SPL.

#### 2.2.3. Questionnaire

The questionnaire (Appendix A), adapted from the one originally developed by Pedersen and Waye [21] was organized in three sections with distinct types of questions. In Section I, the questions aimed at assessing how the respondents reacted to their environment, and detecting any respondent bias that could affect and skew the answers in Section II. The questions in Section II aimed at assessing respondents’ perception of WT noise. Finally, Section III included questions regarding personal data of the respondents, as well as the data on the implementation of mitigation measures in their dwellings, either before or after the installation of the wind farm. The questionnaire was administered by the researchers to the inhabitants of the four villages under study.

The questionnaire targeted the inhabitants of the villages located close to the WT and it was applied to all the people that were living there and available to respond to the questionnaire. As the villages included in the study were quite small and dispersed, the questionnaires were distributed in person, by hand, to adult residents in dwellings potentially exposed to WT noise, who showed willingness to participate in it. In the beginning of the interviews, its nature and objectives were thoroughly explained to the potential respondents and their informed consent obtained. Given that the real number of the villages’ inhabitants at the time the questionnaire was presented was unknown, no answer rates were computed in this study.

### 2.3. Statistical Methods

For statistical purposes, the preceding conceptual framework was operationalized through the following path, or two-equation structural model:

$$R_{i}=f_{1}\left({\mathrm{SPL}}_{i},\;A_{i},\;X_{1i}\right)$$

$$A_{i}=f_{2}\left({\mathrm{SPL}}_{i},\;X_{2i}\right)$$

where R_i_ is the binary response of the ith individual concerning whether or not she/he has spent or has considered spending resources on house retrofitting (“revealed information”); A_i_ is the binary response of the ith individual concerning whether or not she/he feels annoyed or very annoyed with wind turbine noise; SPL_i_ is the actual wind turbine sound pressure level outside of the dwelling of the ith respondent; X_1i_ and X_2i_ are vectors of control variables thought to affect R_i_ and A_i_, respectively.

In the statistical literature, this model is often labelled a *recursive* (or *triangular*) simultaneous equation model [22], and is standard practice to estimate (consistently and efficiently) two-equation structural models through the application of ordinary least squares to each equation separately. Three key features of the present analysis, however, render the standard estimation approach inappropriate. In fact: (i) the primary response variable of interest, R_i_, is a binary variable; (ii) one of the important covariates in the first equation, A_i_, is likely to be jointly determined with the response variable R_i_, being a binary variable as well; and, (iii) the assessment of the overall impact of SPL_i_ on R_i_ requires estimation of both its direct effect as measured in the first equation, and its indirect effect as measured through the effect of A_i_ on R_i_, which is attributable only to changes in SPL_i_.

The first of these features requires the use of a suitable modeling technique for binary dependent variables, such as a probit model. Briefly, the probit model estimates the probability that an event occurs (e.g., that R_i_ takes the unit value) through the construction of an appropriate likelihood function estimable by standard maximum likelihood procedures [23]. The second feature mandates the use of simultaneous equations or structural modeling techniques, whereby the two equations above are jointly estimated. Because A_i_ is a dependent variable in the second (mediating) equation and a predictor of R_i_ in the first (main) equation, a system of equations exists. It is well-known that with such a system of equations, estimating each equation separately is appropriate only if the omitted factors (i.e., the counterparts to error terms in standard linear regression models) in the two equations are uncorrelated. This condition, however, is violated if there is an overlap in unobserved characteristics that determine both A_i_ and R_i_.

Consider, for example, any unmeasured characteristic (i.e., a covariate not included in X_1_ and X_2_ above) that increases individuals’ propensity to feel more annoyed with WT noise, but simultaneously less inclined or less able to incur in retrofitting actions. Because this characteristic is not explicitly included in the set of explanatory variables, its effect on A_i_ and R_i_ gets subsumed into the error terms of both equations. In particular, the error term in the second (first) equation will take higher (lower) values when this characteristic is present. Because the two error terms will move together, depending on the presence of this characteristic, they will be (negatively, in this example) correlated. Importantly, this example illustrates that a consequence of such across equation correlation between the error terms is that the explanatory (mediating) variable A_i_ will correlate with the error term in the first equation. In fact, if the error terms are correlated, and given that the error term in the second equation partly determines A_i_, then A_i_ is also (negatively) correlated with the error term in the first equation. In this case A_i_ will capture not only the true effect of being annoyed with WT noise but also the effect on R_i_ of having this unobservable characteristic. Furthermore, given the negative correlation under consideration, the example illustrates that single estimation of the first equation will suppress any expected positive effect of A_i_ on R_i_, leading to the empirical incorrect conclusion that the mediating effect is weaker than it actually is or that no mediating effect exists when it actually does. The reverse is, of course, also possible, i.e., the existence of positive correlation leading to the conclusion that mediation exists when it actually does not.

Structural modeling techniques solve this problem by explicitly allowing the errors terms across equations to correlate in the model specification, and using full information maximum likelihood methods to estimate the equations as a joint system (e.g., [24]). A difficulty in the present application, however, is that the above system is nonlinear given that both A_i_ and R_i_ are dichotomous variables. This aspect renders familiar simultaneous equations techniques inappropriate. Intuitively, a suitable procedure to address this problem would consist in extending the standard probit model to two equations with correlated error terms. In fact, the so-called bivariate probit model operationalizes this extension (e.g., [22]), and can easily be fit by maximum likelihood methods using, for example, the *Stata* statistical software (StataCorp. 2015. Stata Statistical Software: Release 14. StataCorp. LP, College Station, TX, USA) [25]. This model is implemented assuming that the error terms have a bivariate standard normal joint distribution with correlation *ρ*, and a likelihood-ratio test can be used to test the hypothesis that the bivariate probit model fits the data better than single-equation univariate probit models. This amounts to testing the null hypothesis that *ρ* = 0 against a two-sided alternative; rejection of the null means that joint estimation of the equations is required, whereas failure to reject the null means that the two errors are independent, so that separate estimation of the two structural equations provides consistent estimates of the model’s parameters.

Even if the latter turns out to be true (i.e., *ρ* is not statistically different from zero), this remains a simultaneous equation model, and the third feature above suggests that joint estimation is still warranted (eventually with *ρ* constrained to equal zero). As has been widely documented [22], the coefficients in a univariate probit model do not measure the marginal effect of the associated explanatory variables on the mean response of the dependent variable, as given by the probability that it takes the unit value. In order to compute marginal effects in this model, one must scale the coefficient by the partial derivative of the expression for the probability that the dependent binary variable takes the unit value with respect to the relevant explanatory variable. While this approach yields the marginal effects of the explanatory variables in the second equation (determining A_i_), it fails to measure the total marginal effects of the explanatory variables in the first equation (determining R_i_). Although the computation of these marginal effects in the bivariate probit model is fairly complex [26], it relies on a simple concept: the total marginal effect of a change in a variable in the R_i_ equation must account for the *direct* effect of a change in that variable on the probability that R_i_ equals one, and for the *indirect* effect of the change in that variable on the probability that A_i_ equals one in the second equation. This, in turn, affects the probability that R_i_ equals one in the first equation. Therefore, the total effect of a marginal change in a variable in the first equation is a sum of these two terms, and the delta method [27] can be used to compute the standard error for this sum in order to assess the statistical significance of the overall marginal effect.

Finally, a concern that arises when estimating a system of equations such as those entailed in the bivariate probit model is that the system must be identified (i.e., there must be enough restrictions in the system to obtain numerical estimates of the structural parameters). As shown by Maddala [23] (p. 122), the conditions for identification in the present model imply that the error terms are independent, or else that there is at least one explanatory variable in the second equation (i.e., in X_2i_) not included amongst the explanatory variables in the first equation (i.e., in X_1i_). The first condition entails “fixing” the error terms across equations to zero, which, in the context of the present study, is something that should be tested rather than imposed by assumption. The second condition, and the route adopted in the present study, entails finding at least one variable that, on conceptual grounds, directly affects A_i_ but does not directly affect R_i_ and include (exclude) that variable in the second (first) equation. These procedures, therefore, allow us to quantify and test for the full/partial mediation hypotheses while accounting for the possibility that the error terms across equations correlate within the context of a system of binary dependent variables.

## 3. Results and Discussion

### 3.1. Overview of Variables Used in the Analyses and Descriptive Statistics

Detailed information concerning the sound measurements is synthesized in Table 1. As previously indicated, noise measurements were carried out at eight different locations, designated by the initials of the village name (“CD”, “L”, “VC” and “VP”) followed by “high” and “low”, corresponding to distinct points in each village, at a high and a low location, respectively. Although the registered WT SPL in Table 1 are relatively lower than those reported in other studies (for example, [5,28]), it should be noted that the results from different studies cannot be directly compared with one another due to different measurement conditions and possibly different weather situations at the time of the measurements.

The analyses carried out in the present study encompass several types of variables, including variables related to sound and annoyance (noise perception), and to the respondents’ opinion about WT, the dwelling characteristics, and the respondents’ personal characteristics. A rigorous analysis of the representativeness of the sample is not possible due to lack of information. However, comparing the sample composition with the latest census information available for the biggest village considered in the study (Varzea Cova), it is possible to conclude that the former compares well with the population with respect to age, percentage of females, and schooling years (in 2011, the average age was 48.86, the average number of schooling years was 4.96%, and 51.2% of the inhabitants were female). The definition of the variables used in the analyses as well as the descriptive statistics (means and standard deviations) both for the overall sample and stratified by individuals’ response to the revealed information question are shown in Table 2.

The overall sample is composed of 80 observations, 29 of which correspond to individuals that did spend or have considered spending resources in retrofitting their houses (R = 1), and 51 belonged to the group that did not spend or did not considered spending such resources (R = 0). A number of differences between these two groups of respondents can be detected. Namely, the proportion of individuals annoyed by wind turbine noise both inside (indoors) and outside (outdoors) their dwelling is higher amongst those who spent or have considered spending resources on house retrofitting (52% and 59% in group R = 0, compared to 22% and 20% in group R = 1, indoor and outdoor annoyance, respectively). The proportion of individuals holding negative opinions about WT (i.e., that they are inefficient, unnecessary, and/or have a negative impact on the landscape) is also higher amongst the latter group of individuals. In addition, WT are visible from the dwelling of 76% of the respondents who have not spent nor considered spending resources on house retrofitting, but they are visible from the dwelling of all of the individuals who responded affirmatively to the revealed information question. Likewise, the proportion of individuals reporting being sensitive to noise is higher amongst the latter group of individuals. Conversely, the proportion of individuals benefiting economically from the WT is substantially lower amongst this group.

### 3.2. Statistical Determinants of Noise Annoyance and Local Residents’ Decision to Take Action

The statistical analyses herein consider the *indoors* and *outdoors* reported annoyance separately since these two annoyance metrics were collected from the applied questionnaire and because it is possible that they can be significantly different. For example, Pedersen and Waye [20] report that only a low number of respondents were annoyed indoors by wind turbine noise. Conversely, Hubbard and Sheppard [29] and Thorne [30] state that people exposed to wind turbine noise inside buildings experience a very different acoustic environment than do those outside, and may be more disturbed by the noise inside their homes than they would be outside namely because the acoustic energy from wind turbines generates structural vibrations and is capable of resonating houses.

In each case, selection of the independent variables in the noise annoyance equation is determined by theoretical considerations and previous research findings. Annoyance due to wind turbine noise is thought to depend on the intensity (SPL) of the sound produced by WT to which individuals are exposed to, and on a number of attitudinal, situational and individual factors. In particular, some research findings suggest that noise-induced annoyance is positively influenced by negative opinions about the source itself [31,32]. Annoyance has also been found to be positively correlated with WT visibility from the respondents’ dwellings and with a negative attitude towards the visual impact of WT on the landscape [33,34,35]. Likewise, visibility and distance from the noise source have been found significant by other authors [36]. This negative attitude is enhanced when the landscape is considered of high aesthetic quality [37]. In addition, noise annoyance has also been shown to depend on terrain and urbanization [38]. Other situational factors possibly influencing annoyance responses are the age of the individuals’ dwellings and the topography between the WT and the location of the dwellings. Housing conditions may affect sound transmission and have been previously found to be negatively correlated with the health perception of their residents [39]. As with housing conditions, topography has the potential to alter the auditory sensation and perception of sound pressure levels by, for example, modifying sound propagation, its variability, and/or the sound quality of turbine noise [40].

It should be acknowledged that previous studies have also shown that many other acoustic factors (not considered in the current study) may also play a role on annoyance, especially noise characteristics such as its spectral content or amplitude modulation. In the same way, the noise source visibility issue can be framed in the context of a wider analysis, with emphasis on the way it is perceived and understood by the individual, the so-called soundscape analysis. Previous research in this domain has demonstrated that soundscapes can be described by other equally important descriptors, beyond noise annoyance, e.g., the pleasantness, perceived affective quality, restorativeness, soundscape quality and appropriateness, to name a few [41]. Due to the adopted approach in this study, it should be recognized that the concepts of noise annoyance and the broader concept of soundscape were not fully explored here.

Moreover, evidence shows that annoyance prevalence is also related to personal characteristics of the respondents such as their noise sensitivity, age, and economic conditions, including the possibility to benefit economically from WT [33]. Relatedly, a recent study concluded that support of commercial wind energy depends largely on a belief that wind farms will provide economic benefits to the community [42].

With the exception of two identifying restrictions, these attitudinal, situational and individual factors are also included as control variables in the revealed information equation. The first natural identifying restriction is wind turbine visibility from the respondents’ dwellings, since it does not vary with their response to the revealed information question. Similarly, the second identifying restriction is the respondent’s opinion concerning the visual impact of WT on the landscape. Maximum likelihood estimates of the parameters of the bivariate probit models are reported in Appendix B, and indicate that, taken together, the included explanatory variables are statistically significant determinants of the considered dependent variables. In addition, the estimate of *ρ* is negative and statistically different from zero in both the *Outdoors* and *Indoors* model, implying that the unobserved factors affecting annoyance responses are negatively correlated with those affecting revealed information responses, which vindicates *per se* the use of the structural estimation techniques adopted in the present analyses.

The estimated marginal effects of the explanatory variables in the two equations of the *Indoors* model are given in Table 3. The results show that, ceteris paribus, a unit SPL increase enhances the probability of being annoyed by WT noise by 29.3 percentage points (pp). The results also show that annoyance mediates the relationship between SPL and noise reaction as assessed by revealed information responses. In particular, individuals who feel annoyed by wind turbine noise are 38.9 pp more likely to spend resources on house retrofitting than their counterparts who are not annoyed by it at comparable SPL. Noise-induced annoyance, however, does not fully mediate the relationship between sound intensity and noise reaction. In fact, SPL exert an independent positive effect on the likelihood that individuals spend resources on house retrofitting. The total effect of a unit SPL increase on this likelihood is estimated at 23.2 pp, where 9.3 pp is the direct effect on the likelihood itself, and 13.9 pp is the indirect effect from the probability of being annoyed attributable only to the unit increase in SPL.

The general opinion of the respondents about WT does not seem to have a significant influence in the reported noise annoyance or in the probability to spend more resources improving their houses. However, the results indicate that those respondents who think that WT are inefficient have a higher probability of being annoyed by noise and, through this effect, to be more willing to spend resources on their houses. Actually, individuals who think that WT are inefficient or very inefficient are 73.1 pp more likely to feel annoyed than those who think otherwise, and this also has an indirect effect on the probability to invest in the houses which is 31.1 pp higher for these individuals.

Falling under the heading of “Dwelling characteristics”, all the variables intended to capture situational factors exert a positive and significant effect on self-reported noise annoyance, and through this effect, on the likelihood that individuals spend resources on house retrofitting. In fact, and in line with previous findings by Pedersen [3] and Pedersen and Larsman [43], the results indicate that WT visibility from the respondents’ dwellings increases the probability of being annoyed by WT noise by 58.2 pp. Likewise, all else the same, respondents living in dwellings located at a “high” point in the village are 57.3 pp more likely to feel annoyed by WT noise than their counterparts living in dwellings located at a “low” point in the village. This result provides strong empirical evidence to the conjecture (e.g., [38,40]) that topography impacts the auditory sensation and perception of sound pressure levels, a finding that to the best of our knowledge has not been previously clearly empirically corroborated. The overall effect of WT visibility and topography on individuals’ willingness/necessity to spend resources on house retrofitting is estimated at 21.9 pp and 72.5 pp, respectively. *Ceteris paribus*, the effect of the dwellings’ age on self-reported noise annoyance, albeit statistically significant, is small in magnitude, with a unit increase in this variable leading to an increase in the probability of annoyance by 0.7 pp, and to an indirect increase on the likelihood of investing resources on house retrofitting by 0.2 pp. The results also show that both the direct and the overall effect of this variable on house retrofitting efforts are statistically insignificant, a finding that is somewhat surprising given that one would expect that older houses would need more intervention.

Inspection of the results concerning the effects of respondents’ personal characteristics reveals that, ceteris paribus, none of the included variables has a statistically significant overall impact on house retrofitting efforts. However, all things being equal, female and unemployed individuals are, respectively, 41.3 pp and 30.0 pp significantly more likely to feel annoyed by WT noise than their male and non-unemployed counterparts. Consistent with previous results reported in the literature (e.g., [3,33]), noise sensitivity significantly increases the probability of self-reported annoyance with WT noise by 41.7 pp. Moreover, the results show that, through the effect on annoyance, noise-sensitive individuals are 18.4 pp more likely to incur in house retrofitting efforts than individuals who do not regard themselves as sensitive to noise. As expected from previous research findings, individuals benefiting economically from the noise source are less likely to report being annoyed by it, but unlike results reported by Pedersen et al. [33], this effect is not statistically significant at less than the 5% significance level. Importantly, however, this variable exerts a negative and statistically significant indirect effect on house retrofitting efforts. In fact, the results show that, all else the same, respondents who benefit economically from the WT are 12.6 pp less likely to adopt mitigation measures than those who do not benefit from it, a finding that might reflect attempts from the former to curtail any resentment from their non-profiting neighbors thereby hindering any local objections to these projects.

Turning to the analysis of the *Outdoors* model, the results displayed in Table 4 reveal that the estimates in this model differ little from those in the *Indoors* model both in sign and statistical significance. Importantly, a noticeable exception to this observation concerns the mean impact of SPL on the probability of annoyance perceived outside the dwelling which is here statistically insignificant. This result stands in stark contrast with the finding concerning the mean impact of SPL on the probability of annoyance perceived indoors and provides strong evidence supporting the claim that wind turbine noise causes greater annoyance in the indoor environment which, in turn, may induce sleep disturbance, stress and other illnesses.

Concerning the impact of outdoors annoyance on noise reaction, the results show that individuals who are annoyed by WT noise when spending time outdoors at the dwelling are 57.3 pp more likely to adopt mitigation measures than those individuals who do not experience outdoors annoyance. As previously found in the *Indoors* results, however, noise-induced outdoors annoyance does not fully mediate the relationship between sound intensity as measured by SPL and noise reaction. As seen from the results in Table 4, SPL has an independent positive effect on the likelihood that individuals spend resources on house retrofitting, with a unit SPL increase enhancing this likelihood by 13.5 pp. The total effect of a unit SPL increase on this likelihood is estimated at 17.9 pp, a figure that is lower than the value found in the *Indoors* model due to the lower indirect (i.e., through its effect on outdoor annoyance) impact of SPL increases on house retrofitting decisions, which is here estimated at 4.4 pp.

Taken together, these results are generally in line with existing empirical literature concerning the effects of individuals’ characteristics and their opinions about WT on self-reported annoyance due to wind turbine noise. The results also uncover previously untested relationships between topography, noise-induced annoyance, and noise reaction. In particular, the results suggest that terrain separating windfarms from residential property may attenuate the auditory sensation and perception of SPL at comparable turbine visibility conditions and distance, a finding that may account for the mixed results in the literature concerning the impact of WT noise measured in different locations. Furthermore, our results provide the first corroborative evidence for the proposition that people are more disturbed by wind turbine noise inside their homes than they are outside. Notwithstanding, the results also show that annoyance felt both at indoors and outdoors settings mediates the relationship between SPL and noise reaction. Importantly, a key finding from the results in Table 3 and Table 4 is that noise-induced annoyance does not fully mediate the relationship between sound intensity and noise reaction, with SPL acting as an independent contributor to individuals’ decision to adopt mitigation measures.

## 4. Conclusions

Wind farm projects are frequently opposed by local communities on the grounds that residents develop detrimental physiological and psychological symptoms associated with wind turbine noise. Indeed, noise-induced annoyance presently constitutes the primary response variable used in social surveys to evaluate the health effects of noise on people living close to wind turbines. While useful, self-reported annoyance gives us only a relatively simple sense of how people feel about the noise generated by wind turbines. Moreover, authorities’ adoption of precautionary and/or compensation measures is, in most cases, based on revealed preference arguments-actions that reveal local residents’ perceptions of how grave this environmental hazard is. In particular, individuals’ willingness to perform retrofitting improvements in their houses to improve sound isolation is taken as an indication of both their conviction regarding the causal relationship between noise and health outcomes, and the intensity of their noise-induced suffering.

In this paper, we provide a novel research approach to obtaining behavior-based evidence linking wind turbine noise and noise-induced suffering. Specifically, we propose a novel conceptual framework linking WT sound pressure levels, individuals’ self-reported annoyance, and their revealed or stated willingness to improve their dwellings. In this framework, WT sound pressure levels may directly affect individuals’ decision to implement mitigating actions and/or may indirectly influence that decision through the level of annoyance felt. The proposed framework is empirically evaluated through the application of appropriate structural modeling techniques, and using data collected at a Portuguese wind farm located in a mountain area. In addition, we also address the important challenge of differentiating individuals’ noise-induced annoyance when inside their dwellings from that felt when outside their dwellings at the same sound levels. Although both perceptions may impact individuals’ well-being and subsequent actions, the former is more clearly associated with adverse health effects such as sleep disturbance and psychological distress. Thus, this study offers two significant advances over previous studies, which have mostly been solely based on subjective annoyance metrics, and evaluated either at indoors or outdoors settings from different respondents.

The key finding from this study is that exposure to wind turbine sound significantly impairs individuals’ well-being, because it strongly affects their decision to spend, or consider spending, resources in retrofitting their houses. This effect occurs directly as revealed by exposed individuals’ direct behavioral response to wind turbine sound levels, and not just with noise-induced annoyance acting as mediator in that relationship. Thus, although noise annoyance constitutes an adverse effect in itself and is also an indicator of other possible damaging health effects, the results in this study show that more objective data can be useful when assessing the impact of wind turbine sound on individuals’ health or well-being, such as individuals’ willingness to spend resources to avoid health negative effects or well-being deterioration. The findings of this paper are also relevant at policy level. Firstly, the observed responses provide evidence that the well-being implications of wind farms’ installation may be significant. Secondly, because the observed actions entail financial consequences, they could also inform compensation policies for home owners related to costs arising from measures taken to solve or mitigate the undesired sound effects.

## Figures and Tables

**Table 1:** Summary of noise measurements in the selected villages.

Measurements	Topography and Location							
	High				Low			
	CD	L	VC	VP	CD	L	VC	VP
Mean LAeq-in dB (A)	45.3 (3.6)	42.6 (3.1)	46.2 (1.0)	41.4 (2.4)	46.2 (5.8)	47.6 (3.8)	46.8 (3.9)	48.0 (5.0)
No. measurements	9	5	3	5	4	6	3	4

Note: Standard deviations are in parentheses. L: Lagoa, CD: Campo Dianteiro, VC: Várzea Cova, VP: Vila Pouca.

**Table 2:** Definition of variables and descriptive statistics.

Variable	Revealed Information		Overall	Description
	R_i_ = 0	R_i_ = 1		
*Sound and Annoyance*				
Sound (Sound Pressure Level)	45.22 (2.40)	45.85 (2.13)	45.45 (2.32)	Equivalent continuous Sound Pressure Level (LAeq, in dBA)-SPL
Indoors annoyance	0.22	0.52	0.33	Binary variable, 1 if annoyed or very annoyed, 0 otherwise
Outdoors annoyance	0.20	0.59	0.34	Binary variable, 1 if annoyed or very annoyed, 0 otherwise
*Opinion about Wind Turbines*				
Efficient	0.47	0.38	0.44	Binary variable, 1 if efficient or very efficient, 0 otherwise
Inefficient	0.10	0.31	0.18	Binary variable, 1 if inefficient or very inefficient, 0 otherwise
Necessary	0.55	0.38	0.49	Binary variable, 1 if necessary or very necessary, 0 otherwise
Unnecessary	0.18	0.38	0.25	Binary variable, 1 if unnecessary or very unnecessary, 0 otherwise
Landscape positive	0.37	0.38	0.38	Binary variable, 1 if positive or very positive impact, 0 otherwise
Landscape negative	0.14	0.28	0.19	Binary variable, 1 if negative or very negative impact, 0 otherwise
*Dwelling characteristics*				
Visibility	0.76	1.00	0.85	Binary variable, 1 if WT visible from the dwelling, 0 otherwise
Dwelling age	42.94 (42.62)	45.90 (24.21)	44.01 (36.87)	Number of years since the since the dwelling was built
Topography high	0.41	0.41	0.41	Binary variable, 1 if dwelling is located at a “high” point in the village, 0 otherwise
*Personal characteristics*				
Noise sensitivity	0.55	0.69	0.60	Binary variable, 1 if sensitive to noise, 0 otherwise
Female	0.51	0.52	0.51	Binary variable, 1 if female, 0 otherwise
Age	48.90 (19.48)	48.79 (19.33)	48.86 (19.30)	Respondent’s age, in years
Education	5.96 (3.55)	5.97 (3.35)	5.96 (3.46)	Number of years of schooling
Unemployed	0.10	0.17	0.13	Binary variable, 1 if unemployed, 0 otherwise
Economic benefits	0.22	0.07	0.16	Binary variable, 1 if benefiting economically from the turbines, 0 otherwise
Sample size	51	29	80	Number of respondents

Note: R_i_ takes the unit value if the respondent revealed spending resources on house retrofitting. Standard deviations are in parentheses.

**Table 3:** Estimated marginal effects: indoors model.

Variable	Revealed Information									Annoyance-Indoors		
	Direct				Indirect			Total		Direct		
	Estimate	SE	*p*-Value	Estimate	SE	*p*-Value	Estimate	SE	*p*-Value	Estimate	SE	*p*-Value
*Sound and annoyance*												
Sound (SPL)	0.093	0.043	0.031	0.139	0.049	0.005	0.232	0.073	0.001	0.293	0.091	0.001
Annoyance	0.389	0.086	0.000				0.389	0.086	0.000			
*Opinion about WT*												
Efficient	0.057	0.104	0.588	0.085	0.053	0.111	0.142	0.133	0.288	0.178	0.121	0.141
Inefficient	0.116	0.159	0.465	0.311	0.109	0.004	0.427	0.222	0.054	0.731	0.201	0.000
Necessary	−0.006	0.125	0.959	0.034	0.050	0.501	0.028	0.153	0.857	0.096	0.131	0.464
Unnecessary	0.180	0.132	0.171	0.057	0.063	0.362	0.237	0.170	0.161	0.002	0.151	0.988
Landscape positive				−0.048	0.048	0.318	−0.048	0.048	0.318	−0.128	0.124	0.301
Landscape negative				0.008	0.050	0.869	0.008	0.050	0.869	0.022	0.133	0.868
*Dwelling characteristics*												
Visibility				0.219	0.107	0.040	0.219	0.107	0.040	0.582	0.232	0.012
Dwelling age	−0.001	0.001	0.549	0.002	0.001	0.037	0.001	0.002	0.522	0.007	0.002	0.000
Topography high	0.388	0.184	0.036	0.337	0.133	0.011	0.725	0.258	0.005	0.573	0.265	0.031
*Personal characteristics*												
Noise sensitivity	0.087	0.104	0.401	0.184	0.090	0.042	0.271	0.157	0.084	0.417	0.200	0.037
Female	−0.022	0.104	0.831	0.148	0.079	0.062	0.126	0.148	0.395	0.413	0.169	0.015
Age	−0.001	0.004	0.825	0.003	0.002	0.163	0.002	0.005	0.652	0.008	0.005	0.083
Education	0.013	0.017	0.435	0.001	0.009	0.904	0.014	0.021	0.500	−0.008	0.025	0.748
Unemployed	−0.034	0.151	0.819	0.102	0.068	0.133	0.068	0.192	0.726	0.300	0.142	0.035
Economic benefits	−0.143	0.142	0.314	−0.126	0.048	0.008	−0.269	0.169	0.110	−0.217	0.121	0.074

Note: SE is the estimate standard error.

**Table 4:** Estimated marginal effects: outdoors model.

Variable	Revealed Information									Annoyance-Outdoors		
	Direct			Indirect			Direct			Indirect		
	Estimate	SE	*p*-Value	Estimate	SE	*p*-Value	Estimate	SE	*p*-Value	Estimate	SE	*p*-Value
*Sound and Annoyance*												
Sound	0.135	0.039	0.001	0.044	0.016	0.007	0.179	0.045	0.000	0.011	0.040	0.782
Annoyance	0.573	0.072	0.000				0.573	0.072	0.000			
*Opinion about WT*												
Efficient	0.042	0.090	0.644	0.022	0.031	0.463	0.064	0.106	0.546	0.029	0.094	0.755
Inefficient	0.006	0.131	0.961	0.082	0.058	0.156	0.088	0.165	0.592	0.238	0.155	0.125
Necessary	0.063	0.126	0.616	−0.039	0.045	0.385	0.024	0.155	0.877	−0.172	0.111	0.121
Unnecessary	0.253	0.137	0.064	0.008	0.054	0.876	0.261	0.174	0.134	−0.201	0.120	0.095
Landscape positive				0.049	0.040	0.228	0.049	0.040	0.228	0.145	0.104	0.163
Landscape negative				0.029	0.043	0.500	0.029	0.043	0.500	0.085	0.120	0.479
*Dwelling characteristics*												
Visibility				0.107	0.046	0.019	0.107	0.046	0.019	0.317	0.130	0.015
Dwelling age	0.000	0.001	0.720	0.001	0.001	0.310	0.001	0.002	0.535	0.001	0.002	0.444
Topography high	0.372	0.173	0.031	0.193	0.072	0.007	0.565	0.208	0.007	0.242	0.166	0.145
*Personal characteristics*												
Noise sensitivity	−0.024	0.101	0.811	0.096	0.048	0.047	0.072	0.132	0.586	0.306	0.086	0.000
Female	−0.061	0.092	0.507	0.051	0.038	0.178	−0.01	0.116	0.930	0.205	0.094	0.028
Age	−0.001	0.003	0.856	0.001	0.001	0.334	0.0007	0.004	0.869	0.004	0.004	0.213
Education	0.009	0.016	0.572	0.012	0.006	0.046	0.021	0.020	0.281	0.028	0.017	0.093
Unemployed	0.014	0.138	0.920	0.001	0.049	0.976	0.015	0.172	0.929	−0.008	0.118	0.946
Economic benefits	−0.042	0.126	0.743	−0.159	0.038	0.000	−0.201	0.146	0.169	−0.435	0.117	0.000

Note: SE is the estimate standard error.

## Appendix A. Questionnaire on Wind Turbine Noise Perception (Adapted from Pedersen & Waye [21])

### Section I

1. How satisfied are you with your living environment? (Very satisfied, satisfied, not so satisfied, not satisfied, not at all satisfied)
2. Have there been any changes to the better in your living environment/municipality during the last years? (No, yes! If yes, state what changes)
3. Have there been any changes to the worse in your living environment/municipality during the last years? (No, yes! If yes, state what changes)
4. State for each of the following factors your degree of nuisance when you spend time outdoors at your dwelling: odors from industries (if applicable), odors from fertilizers, insects, fans, noise from wind turbines, railway noise (if applicable), road traffic noise, lawn mowers noise. (Do not notice, notice but not annoyed, slightly annoyed, rather annoyed, very annoyed)
5. State for each of the following factors your degree of nuisance when you are indoors in your dwelling: odors from industries (if applicable), odors from fertilizers, insects, fans, noise from wind turbines, railway noise (if applicable), road traffic noise, lawn mowers noise. (Do not notice, notice but not annoyed, slightly annoyed, rather annoyed, very annoyed)
6. How would you describe your sensitivity to the following factors: air pollution? Odors? Noise? Littering? (Not sensitive at all, slightly sensitive, rather sensitive, very sensitive)

### Section II

1. Are you able to see any wind turbine from your dwelling or your garden? (Yes, No)
2. What is your opinion on the impact of wind turbines on the landscape? (Very positive, positive, neither positive nor negative, negative, very negative)
3. State for each of the following factors how you are affected by wind turbines when you are indoors in your dwelling: shadows from rotor blades? Reflections from rotor blades? Sound from rotor blades? Sound from machinery? Changes in the view? (Do not notice, notice but not annoyed, slightly annoyed, rather annoyed, very annoyed)
4. If you are annoyed by noise, shadows and/or reflections from wind turbines, state how often does this happen? (Never/almost never, a few times per year, a few times per month, a few times per week, daily/almost daily)
5. If you can hear the sound generated by wind turbines, how would you describe that sound and what is your reaction to it: tonal, pulsating/throbbing, swishing, whistling, lapping, scratching/squeaking, low frequency, resounding. (Do not notice, notice but not annoyed, slightly annoyed, rather annoyed, very annoyed)
6. How would you characterize the sound from wind turbines in the following special occasions: when the wind blows from the turbines towards my dwelling, when the wind blows towards the turbines, when the wind is week, when the wind is strong, in warm summer nights? (Less clearly heard, more clearly heard, no difference, do not know)
7. Are you annoyed by sound from wind turbines during any of the following activities: relaxing outdoors, barbecue nights, taking a walk, while gardening, other outdoor activities? (Do not notice, notice but not annoyed, slightly annoyed, rather annoyed, very annoyed)
8. Do you own any wind turbines, or are you involved in wind energy? (No, but I rented a space for wind turbines installation, yes I own one or more turbines, yes I own shares of a company that builds/installs wind turbines, yes I own shares of a company that generates electricity from turbines)
9. What is your general opinion on wind turbines? (Very positive, positive, neither positive nor negative, negative, very negative)
10. Please mark the adjectives that you think are adequate for wind turbines (efficient-inefficient, environmentally friendly-environmentally harmful, unnecessary-necessary, aesthetically ugly-aesthetically nice, threatening-harmonious)

### Section III

1. Personal data
  17.1. Gender
  17.2. Age
  17.3. Educational background
  17.4. Employment situation
2. Data about respondents’ dwelling
  18.1. Year the dwelling was built?
  18.2. If the dwelling construction was later than the wind farm installation, indicate if any mitigation measures in its characteristics were adopted (No, yes. Which?)
  18.3. If the dwelling construction was earlier than the wind farm installation, indicate if any mitigation measures in the dwelling characteristics were adopted (No, yes. Which? No, but already felt that need *)
  * In this case, please indicate what measures you feel need to be adopted.

## Appendix B

**Table A1:** Estimated bivariate probit models: outdoors and indoors annoyance.

Variable	Outdoors						Indoors					
	Revealed Information			Annoyance			Revealed Information			Annoyance		
	Coeff.	SE	*p*-Value	Coeff.	SE	*p*-Value	Coeff.	SE	*p*-Value	Coeff.	SE	*p*-Value
*Sound and Annoyance*												
Sound (SPL)	0.624	0.193	0.001	0.082	0.207	0.692	0.341	0.174	0.050	1.872	0.732	0.011
Annoyance	2.618	0.433	0.000				1.526	0.337	0.000			
*Opinion about WT*												
Efficient	0.184	0.414	0.656	0.168	0.505	0.739	0.200	0.395	0.613	1.139	0.789	0.149
Inefficient	0.012	0.597	0.984	1.242	0.865	0.151	0.436	0.620	0.482	4.727	1.688	0.005
Necessary	0.299	0.577	0.605	−0.945	0.611	0.122	−0.009	0.483	0.986	0.552	0.929	0.553
Unnecessary	1.190	0.621	0.055	−1.035	0.658	0.116	0.700	0.523	0.181	0.008	0.976	0.994
Landscape positive				0.829	0.574	0.149				−0.898	0.786	0.253
Landscape negative				0.486	0.625	0.437				0.121	0.854	0.887
*Dwelling characteristics*												
Visibility				1.625	0.705	0.021				3.852	1.560	0.014
Dwelling age	0.002	0.006	0.730	0.006	0.008	0.490	−0.003	0.006	0.566	0.044	0.015	0.003
Topography high	1.734	0.816	0.034	1.378	0.836	0.099	1.429	0.733	0.051	3.534	1.846	0.056
*Personal characteristics*												
Noise sensitivity	−0.088	0.447	0.844	1.633	0.550	0.003	0.327	0.405	0.420	2.739	1.400	0.050
Female	−0.281	0.419	0.503	1.061	0.552	0.054	−0.077	0.402	0.847	2.691	1.273	0.034
Age	−0.002	0.015	0.875	0.022	0.020	0.253	−0.004	0.014	0.766	0.053	0.033	0.112
Education	0.041	0.072	0.571	0.146	0.094	0.120	0.049	0.066	0.464	−0.066	0.144	0.647
Unemployed	0.056	0.633	0.93	−0.024	0.625	0.970	−0.155	0.579	0.788	1.891	1.010	0.061
Economic benefits	−0.194	0.559	0.728	−2.313	0.745	0.002	−0.557	0.556	0.316	−1.455	0.844	0.085
*Constant*	−30.933	9.395	0.001	−9.828	9.557	0.304	−17.327	8.379	0.039	−100.589	36.749	0.006

Notes: Log-likelihood value for the Outdoors (Indoors) model is −59.032 (−56.384); Wald test for the null hypothesis that all coefficients are zero in the Outdoors (Indoors) model has a χ^2^ value of 65.93 (50.62) with 30 df, implying a *p*-value equal to 0.0002 (0.0107). In both models, the estimated correlation between the error terms, *ρ*, is negative (≅−0.99); Likelihood-ratio test that *ρ* equals zero in the Outdoors (Indoors) model has a χ^2^ value of 7.82 (5.91) with 1 df, implying a *p*-value equal to 0.0052 (0.0151).
